# 553 Nursing-Led Education and Interventions to Reduce Fungal Infections on a Burn Unit

**DOI:** 10.1093/jbcr/irae036.187

**Published:** 2024-04-17

**Authors:** Hannah Thielges, Abigail Rhodes, Sophia A Marvin, Trevor M Kraeger, Megan M Rasmussen, Clea Gade, Eleanor Thibodeau, Bradley Rogers, Iniabasi Nkanga Mitchell, Katelyn Rufi

**Affiliations:** Regions Hospital, Saint Louis Park, MN; Regions Hospital, Minnetonka, MN; Regions Hospital, White Bear Lake, MN; Regions Hospital Burn Center, Woodbury, MN; Regions Hospital, Saint Paul, MN; Regions Hospital Burn Center, Fridley, MN; Regions Hospital Burn Center, St. Paul, MN; Regions Hospital, Brooklyn Park, MN; Regions Hospital, Saint Louis Park, MN; Regions Hospital, Minnetonka, MN; Regions Hospital, White Bear Lake, MN; Regions Hospital Burn Center, Woodbury, MN; Regions Hospital, Saint Paul, MN; Regions Hospital Burn Center, Fridley, MN; Regions Hospital Burn Center, St. Paul, MN; Regions Hospital, Brooklyn Park, MN; Regions Hospital, Saint Louis Park, MN; Regions Hospital, Minnetonka, MN; Regions Hospital, White Bear Lake, MN; Regions Hospital Burn Center, Woodbury, MN; Regions Hospital, Saint Paul, MN; Regions Hospital Burn Center, Fridley, MN; Regions Hospital Burn Center, St. Paul, MN; Regions Hospital, Brooklyn Park, MN; Regions Hospital, Saint Louis Park, MN; Regions Hospital, Minnetonka, MN; Regions Hospital, White Bear Lake, MN; Regions Hospital Burn Center, Woodbury, MN; Regions Hospital, Saint Paul, MN; Regions Hospital Burn Center, Fridley, MN; Regions Hospital Burn Center, St. Paul, MN; Regions Hospital, Brooklyn Park, MN; Regions Hospital, Saint Louis Park, MN; Regions Hospital, Minnetonka, MN; Regions Hospital, White Bear Lake, MN; Regions Hospital Burn Center, Woodbury, MN; Regions Hospital, Saint Paul, MN; Regions Hospital Burn Center, Fridley, MN; Regions Hospital Burn Center, St. Paul, MN; Regions Hospital, Brooklyn Park, MN; Regions Hospital, Saint Louis Park, MN; Regions Hospital, Minnetonka, MN; Regions Hospital, White Bear Lake, MN; Regions Hospital Burn Center, Woodbury, MN; Regions Hospital, Saint Paul, MN; Regions Hospital Burn Center, Fridley, MN; Regions Hospital Burn Center, St. Paul, MN; Regions Hospital, Brooklyn Park, MN; Regions Hospital, Saint Louis Park, MN; Regions Hospital, Minnetonka, MN; Regions Hospital, White Bear Lake, MN; Regions Hospital Burn Center, Woodbury, MN; Regions Hospital, Saint Paul, MN; Regions Hospital Burn Center, Fridley, MN; Regions Hospital Burn Center, St. Paul, MN; Regions Hospital, Brooklyn Park, MN; Regions Hospital, Saint Louis Park, MN; Regions Hospital, Minnetonka, MN; Regions Hospital, White Bear Lake, MN; Regions Hospital Burn Center, Woodbury, MN; Regions Hospital, Saint Paul, MN; Regions Hospital Burn Center, Fridley, MN; Regions Hospital Burn Center, St. Paul, MN; Regions Hospital, Brooklyn Park, MN; Regions Hospital, Saint Louis Park, MN; Regions Hospital, Minnetonka, MN; Regions Hospital, White Bear Lake, MN; Regions Hospital Burn Center, Woodbury, MN; Regions Hospital, Saint Paul, MN; Regions Hospital Burn Center, Fridley, MN; Regions Hospital Burn Center, St. Paul, MN; Regions Hospital, Brooklyn Park, MN; Regions Hospital, Saint Louis Park, MN; Regions Hospital, Minnetonka, MN; Regions Hospital, White Bear Lake, MN; Regions Hospital Burn Center, Woodbury, MN; Regions Hospital, Saint Paul, MN; Regions Hospital Burn Center, Fridley, MN; Regions Hospital Burn Center, St. Paul, MN; Regions Hospital, Brooklyn Park, MN

## Abstract

**Introduction:**

Fungal infections cause major complications in patients with significant burns who are also hospitalized for more than seven days. In this project, significant burns are defined as meeting this burn unit’s Total Body Surface Area (TBSA) isolation precaution requirements: adult patients with TBSA greater than 20 percent and pediatric patients with TBSA greater than 15 percent. Complications of fungal infections include increased length of stay (LOS) and increased risk of mortality, sepsis, and graft loss (Herndon, 2018). Implementing effective strategies to reduce fungal infections is crucial to improving patient outcomes. At this time, there is limited research regarding how bedside nursing-led initiatives can decrease fungal infections in burn units. The purpose of this project is to determine if bedside nursing-led policy revision followed by nursing-generated education and implementation decreases the rate of fungal infections in burn patients that meet the TBSA isolation precaution requirements compared to infection rates prior to this initiative.

**Methods:**

In this quality improvement project, a group of bedside nurses revised an infection prevention protocol and delivered education to all burn nursing staff regarding the new and improved policies, including Burn TBSA Contact Precautions, Standard Burn Precautions, and Advanced Burn Precautions. Nursing staff were empowered to enforce these new precautions with all other hospital staff and visitors on the Burn Unit. Fungal infection rates were analyzed before and after the policy implementation, educational interventions, and staff empowerment.

**Results:**

Our final analysis will compare data from June 12, 2022 through Dec 12, 2022 to data from June 12, 2023 through December 12, 2023. Our preliminary findings demonstrate 2 out of 11 burn patients (eighteen percent) meeting project criteria developed a fungal infection in 2023 compared to 6 out of 18 eligible patients (thirty-three percent) in the 2022 timeframe. This demonstrates a fifteen percent decrease in the rate of infection of eligible burn patients. Confounding variables during the intervention period include a variety of environmental and supply chain adjustments that were also aimed at decreasing infection risk. These changes occurred in tandem with nursing-led initiatives.

**Conclusions:**

Bedside nurse-driven policy changes, education, and interventions may be an effective way to decrease the rate of fungal infections in patients with significant burn injuries.

**Applicability of Research to Practice:**

This project could demonstrate the efficacy of nurse driven policy development and education in the reduction of fungal infection in burn patients. Research in this area is currently lacking.

